# Training Cognitive Functions Using Mobile Apps in Breast Cancer Patients: Systematic Review

**DOI:** 10.2196/10855

**Published:** 2019-03-19

**Authors:** Laura Vergani, Giulia Marton, Silvia Francesca Maria Pizzoli, Dario Monzani, Ketti Mazzocco, Gabriella Pravettoni

**Affiliations:** 1 Department of Oncology and Hemato-Oncology University of Milan Milan Italy; 2 Applied Research Division for Cognitive and Psychological Science European Institute of Oncology (Istituto di Ricovero e Cura a Carattere Scientifico) Milan Italy

**Keywords:** cognitive impairment, breast cancer, cognitive training, intervention, mobile-based interventions

## Abstract

**Background:**

Breast cancer is an invalidating disease and its treatment can bring serious side effects that have a physical and psychological impact. Specifically, cancer treatment generally has a strong impact on cognitive function. In recent years, new technologies and eHealth have had a growing influence on health care and innovative mobile apps can be useful tools to deliver cognitive exercise in the patient’s home.

**Objective:**

This systematic review gives an overview of the state-of-the-art mobile apps aimed at training cognitive functions to better understand whether these apps could be useful tools to counteract cognitive impairment in breast cancer patients.

**Methods:**

We searched in a systematic way all the full-text articles from the PubMed and Embase databases.

**Results:**

We found eleven studies using mobile apps to deliver cognitive training. They included a total of 819 participants. App and study characteristics are presented and discussed, including cognitive domains trained (attention, problem solving, memory, cognitive control, executive function, visuospatial function, and language). None of the apps were specifically developed for breast cancer patients. They were generally developed for a specific clinical population. Only 2 apps deal with more than 1 cognitive domain, and only 3 studies focus on the efficacy of the app training intervention.

**Conclusions:**

These results highlight the lack of empirical evidence on the efficacy of currently available apps to train cognitive function. Cognitive domains are not well defined across studies. It is noteworthy that no apps are specifically developed for cancer patients, and their applicability to breast cancer should not be taken for granted. Future studies should test the feasibility, usability, and effectiveness of available cognitive training apps in women with breast cancer. Due to the complexity and multidimensionality of cognitive difficulties in this cancer population, it may be useful to design, develop, and implement an ad hoc app targeting cognitive impairment in breast cancer patients.

## Introduction

Cancer is a major public health problem worldwide and the second leading cause of death in the United States. In 2017, Siegel and colleagues [1] reported 1,688,780 new cases and estimated 600,920 deaths. Breast cancer is one of the most common cancers around the world [2]. It is the most common cancer diagnosed in women, affecting about 1 in 8 women in the United States during their lifetime (12.4%), and it is the second most common cause of death by cancer in women [3]. The incidence rate is strictly associated with age: 95% of new cases are registered in women older than 40 years; only 1.5 cases per 100,000 are registered in young women (20 to 24 years). The incidence is higher for older women: 421.3 cases per 100,000 women are registered in women 75 to 79 years old; the median age at diagnosis is 61 years [3]. Prognosis is related to cancer stage: 5-year survival rate is 100% for stage 0 and I, 93% for stage II, 72% for stage III, and 22% for stage IV [3]. In the last 20 years, we have witnessed a decline in overall cancer mortality. This is especially true for breast cancer mortality: the death rate steadily decreased by 38% from 1989 to 2014. This is due especially to decreases in smoking and advances in early detection and treatment [1].

The advances made in breast cancer treatment offer women greater prospects of cure and a better quality of life. However, cancer treatment has some deleterious acute or long-term side effects that impact women’s physical, functional, emotional, financial, and social lives [4]. Along with depression and anxiety [5], cognitive impairment is a short- or long-term outcome or side effect of breast cancer and its treatment and the treatment of other diseases such as stroke [6], HIV and hemophilia [7], and multiple sclerosis [8]. In recent years, empirical evidence has risen regarding significant cognitive impairment following breast cancer treatments in survivor women. The American Cancer Society/American Society of Clinical Oncology Breast Cancer Survivorship Care Guideline [9] showed that 75% and 35% of patients report cognitive impairment during treatment and after treatment, respectively. In addition, in everyday clinical practice, women with breast cancer often complain about cognitive difficulties in different domains of cognition. For example, these cognitive impairments include problems with concentration, executive function, memory [9] and, especially in patients treated with chemotherapy, problems with visual memory, information processing speed, and verbal memory [10]. Cognitive impairments could also reduce the quality of life, lead to distress, and have a negative impact on women’s working, societal, and family life [9].

Cognitive impairment can have multifactorial causes. Runowicz and colleagues [9] reported that insomnia, depression, fatigue, surgery, anesthesia, different type of cancer treatments and cancer itself could cause cognitive impairment. For example, a meta-analysis conducted by Jim and colleagues [11] showed that breast cancer survivors treated with chemotherapy suffer small and limited observed cognitive deficits. This is especially true for the domains concerning verbal and visuospatial ability. However, other difficulties also concern memory and attention deficits due to chemotherapy. These symptoms, generally known as *chemobrain* or *chemofog*, are experienced by a lot of women treated with chemotherapy [4]. Bakoyiannis and colleagues [12] conducted a systematic review on the impact of endocrine therapy on cognitive functions of breast cancer patients. They especially investigated difficulties in 5 cognitive domains: verbal memory, verbal fluency, attention and working memory, motor speed, and psychomotor speed. They concluded that endocrine therapy may alter cognitive functions in these women.

However, the exact mechanism underlying cognitive impairment is not clear. For example, empirical evidence exists on the role of stress and coping styles [13], direct neurotoxic injury, telomere shortening, oxidative stress, cytokine dysregulation, estrogen-mediated effects, genetic polymorphism [14], peripheral proinflammatory cytokines [15], decreased estrogen levels, and structural brain changes [16] in cognitive impairment following cancer treatment.

Moreover, there are uncertainties regarding the nature and magnitude of cognitive impairment in breast cancer patients [17] and also regarding the most effective treatment to target these kinds of cognitive difficulties [9]. In their review on pharmacological and nonpharmacological interventions to manage cognitive alterations after chemotherapy, Chan and colleagues [18] concluded that pharmacological interventions to manage cognitive alterations after chemotherapy for breast cancer are not well supported by current empirical evidence. On the other hand, some kind of cognitive training—computerized cognitive training, cognitive behavioral therapy, memory training, speed of processing training, psychoeducation, Tibetan sound—and physical interventions may be useful. However, further studies are needed in order to provide guidelines and concrete recommendations for clinical practice [18].

Overall, these psychological interventions for cognitive impairment are time- and money-consuming. They may result in a huge burden for patients because they are often delivered in an in-patient context [17]. The development and delivery of home-based or Web-based interventions may have advantages over traditional clinic-based ones. However, there is a scarcity of these kinds of innovative interventions for cognitive training.

Starting with the introduction of the World Wide Web in our daily lives, the internet has increasingly become an essential part of modern living. A recent report by the Pew Research Center states that in the developed nations, the number of people who use the internet or a mobile phone remains high throughout the years (around 86% from 2015 to 2018) and that in the developing world the rate is increasing constantly (from 62% of the population using the internet or owning a mobile phone in 2013 to 64% in 2018) [19]. A common misunderstanding is that the elderly, compared to younger people, are less interested in technology. However, older people are rapidly gaining more interest in the subject [20], and the rate of people aged 65 years and above using the internet grew from 14% in 2000 to 58% in 2015. Internet use for other age groups is growing as well: internet use from 2000 to 2015 increased from 70% to 96% for people aged 18 to 29 years, from 61% to 93% for people aged 30 to 49 years, and from 46% to 81% for people aged 50 to 64 years. These statistics are especially interesting because they could indicate the promise of development of innovative Web-based interventions also targeting elderly people. Traditional health care interventions have been delivered through face-to-face meetings with clinicians. However, eHealth and mHealth uses have increasingly spread in the last decades [21].

The two leading platforms for health-related mobile apps are iOS and Android. As of 2014, more than 100,000 mHealth apps had been released [22,23]. mHealth apps permit real-time and bidirectional interaction with the patient [23]. This transformation brings changes even in the diagnosis and treatment of cancer.

Several mHealth apps have been developed to inform patients; enhance communication, consulting, and symptom self-management and monitoring; and improve health record access and maintenance and clinical decision making [24]. However, little is known about apps directly aimed at improving cognitive functioning in patients reporting cognitive difficulties. No apps have been specifically developed to counteract cognitive difficulties in breast cancer survivors. Thus, we aimed to identify effective apps to train cognitive function and counteract cognitive impairment in both clinical and nonclinical populations. Another aim was to evaluate their efficacy and assess whether they could be also used to counteract cognitive impairment in breast cancer patients. Thus, we focused on their distinctive features in terms of targeted populations and the specific cognitive domains being trained.

## Methods

**Figure 1:**
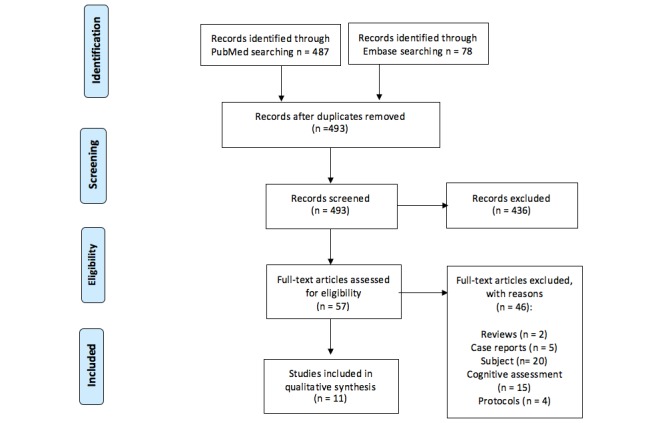
Flowchart of the systematic review.

Our search strategy was designed according to the Preferred Reporting Items for Systematic Reviews and Meta-Analyses guidelines [25]. A flowchart of the systematic review is shown in Figure 1.

To identify articles for our review, we searched PubMed and Embase databases for the terms smartphone, mobile, and app. The searched cognitive domains were memory, attention, concentration, verbal fluency, motor speed, psychomotor speed, problem-solving, executive function, visuospatial function, language, and cognitive control. In particular, the string of terms included:

- Smartphone app or mobile app
- Cognitive function or memory or attention or concentration or verbal fluency or motor speed or psychomotor speed or problem solving or executive function or visuospatial function or language or cognitive control

The search was limited to the English language. No restriction was placed on the year of publication. The search was completed in February 2018.

Two authors independently reviewed the titles and abstracts to identify relevant papers. We extracted only those papers with the main focus on the use of a mobile-based app (mobile phone or tablet-based) to deliver cognitive training. About 10% of the papers were double screened, and disagreements in data extraction were resolved through discussion with a third author.

The second step consisted of a full article screening. We excluded papers that were (1) reviews, case reports, or protocols; (2) abstracts for conferences; or (3) related to apps that assess cognitive functions without training them.

Data collection included information on authors, title, publication date, study aim, sample characteristics, clinical or nonclinical populations, cognitive domains trained, app exercise descriptions, assessment and feedback, app evaluation and efficacy, training with the experimenters, and whether the device was given to the study participants.

## Results

### Studies Selected

A flowchart of the systematic review is shown in Figure 1. We retrieved a total of 493 articles. We selected 11 articles after a full-text screening. We excluded 2 reviews (eg, [21,26]), 5 case reports (eg, [27-31]), and 4 protocols (eg, [32,33]). The other studies were excluded for content reasons, for example: some studies did not use apps to train cognitive functions [22,34-47], and others were excluded because they used apps only to assess—and not to train—cognitive functions [48-58]. Our 11 selected studies focus on the cognitive domains of attention, memory, problem solving, cognitive control, executive function, visuospatial function, and language (reported in Table 1).

### Sample Characteristics

Our search returned 11 studies that include 819 participants. The number of participants in these studies ranged from 9 to 626. Weighted by sample size, the mean age of participants was 36.2 years. However, there is a huge heterogeneity in participant ages among these 11 studies: ages ranged from 50 months to 96 years. In particular, 1 study focused on preschool children with a mean age of 60 months [59], while 4 papers focused especially on older adults with a mean age over 68 years [60-63].

Nearly all of the studies were performed in developed countries. Almost half of the studies (5/11, 45%) were conducted in North America: 4 were performed in the United States [60,61,64,65] and 1 in Canada [63]. Three were conducted in Europe: 2 in Italy [59,66], and 1/11 in Norway [67]. Two studies were conducted in Asia: 1 in Taiwan [62] and 1 in Jordan [68]. Only 1 study was conducted in Oceania (Australia [69]). In addition to this heterogeneity in cultural provenance, the 11 identified studies have a moderate variability in the kind of population in which they evaluated the app for the cognitive training. No studies involved breast cancer patients. Ten of 11 studies involved adults or elderly people. One study focused on adults affected by brain injuries [65], 1 on cognitively impaired patients with multiple sclerosis [66], and 1 on patients with early stages of Alzheimer disease [68]. Three papers focused on healthy elderlies [60-62] while 1 study chose a population of older adults with and without subjective cognitive complaints and mild cognitive impairment [63]. One study focused on adults with mild to moderate depression [64] and 1 on overweight or obese adult [69]. Just 1 paper from our search specified healthy young adults [67]. Only 1 study focused on children with mild to severe language impairments or delays [59]. Inclusion and exclusion criteria were different in all of the studies. Only 3 studies [65,67,69] included control groups.

### Aim

The majority of the selected papers had a primary aim to evaluate the apps in terms of usability, acceptability, feasibility, and user satisfaction [59-61,63,66-68]. Only 2 articles [62,65] chose to describe the development and improved design of the apps. Only 1 paper [64] had as the main aim to assess and improve depressive symptoms by comparing 3 different apps, 1 of which is explicitly aimed at improving depression through a cognitive control exercise. Just 1 study had the explicit aim of investigating the efficacy of the app in improving cognitive functioning [69].

### App Characteristics

App availability on the market was checked by searching in the main app stores (App Store and Google Play); however, none are available to the general public. The same search was conducted on online test catalogs, such as Pearson Assessments or Psychological Assessment Resources Inc, but no apps from the studies were found. In all of the studies, app users were asked to respond with a touchscreen.

**Table 1:** App developers, app names, and cognitive domains being targeted in each study.

Author, year	App name	Attention	Problem solving	Memory	Cognitive control	Executive function	Visuospatial function	Language
Arean and colleagues, 2016 [64]	Project: EVO				✓			
Blackburne and colleagues, 2016 [69]	NoGo				✓			
Bless and colleagues, 2014 [67]	—	✓						
Hill and colleagues, 2015, and Hill and colleagues, 2018 [60,61]	Attention Training Application	✓						
Lorusso and colleagues, 2018 [59]	—							✓
Lu and colleagues, 2017 [62]	Brain Win	✓		✓		✓	✓	✓
Powell and colleagues, 2017 [65]	ProSolv		✓					
Shellington and colleagues, 2017 [63]	Health*e* Brain			✓		✓	✓	
Tacchino and colleagues, 2015 [66]	Cognitive Training Kit (COGNI-TRAcK)			✓				
Zmily and colleagues, 2014 [68]	ADcope – Spaced Retrieval Exercise			✓				

Bless and colleagues [67] explored the feasibility and effectiveness of an app that enables people to train auditory attention. They developed the app in-house, but they did not give it a name. Participating in this study were healthy adults; they were given an iPod touch and asked to perform a consonant-vowel dichotic listening task. According to the forced-attention condition of the standard consonant-vowel dichotic listening paradigm, during this task each participant was presented simultaneously with 2 different syllables via earphones: while one was presented to the left ear, the other was delivered to the right. Syllables, made by consonants and vowels, were read by a male, native Norwegian speaker with constant voice intonation and intensity. Before the beginning of the task, each participant was told via message on the display to pay attention only to the syllable delivered to the left or the right ear. After hearing the syllables, participant had to choose the correct syllable from 6 multiple choice answers. Each training session, about 6 minutes long, included 6 blocks with 5 pairs of syllables. Syllables were presented in 400 to 500 ms with an interval of 4000 ms between each pair. At the end of each session, participants were shown feedback of their correct answers. Results were stored on the device and available to the researcher at the end of the study.

Lorusso and colleagues [59] developed an app to improve semantic competence and structural knowledge in children with learning impairments. It was part of an integrated system. The aim of the study was to evaluate learnability, usability, user satisfaction, and the quality of the interaction in children playing with this integrated system. The app was used on a supplied tablet; it scanned the tag that the experimenter had placed under some toys (plastic animals). When the children scanned the tag under the toys, the app displayed a menu with 5 different activities that were all related to this specific animal. The first was an informative activity in which children were given some interesting information about this specific animal. The second one was a storytelling activity that directly connected to a website with stories and tales related to the animal. The third was a visual activity with several picture and photos of this specific animal. The fourth presented a song strictly related to the chosen animal. The last one was a puzzle activity in which children were asked to put together pieces of a puzzle representing this specific animal. The authors chose all these activities because they enrich semantic knowledge and organization. After the completion of each the task, the child was given feedback by picture or sound. The app also provided a text-to-speech tool to listen to written information.

Characteristics of the included studies and related apps are presented in Multimedia Appendix 1 and 2.

Shellington and colleagues [63] examined the feasibility and utility of an app called HealtheBrain, which was focused on improving visuospatial executive function and working memory. Participants of this study were older adults with or without subjective cognitive complaints and mild cognitive impairment. They received HealtheBrain to improve cognitive functioning through a specific physical exercise called Square Step Exercise. Before the beginning of the exercise, the app offered participants the opportunity to have a tutorial about the use of the app and then to calibrate the step length. The square step exercise had 35 progressive stages and the participants had to start to form the first one. In order to go from 1 level to another, the subject had to complete at least 80% of each task. For each step, participants had to memorize 4 to 8 walking and stepping sequences and repeat them. When they reached the goal, an item was added to their virtual garden and they could get to the next level. They were asked to exercise at least 3 times per week over the next 3 weeks, and subjects were invited to contact the researchers with any question or technical issue.

In 2015, Hill and colleagues [60] developed an app to train attention. More recently [61], they decided to improve it considering the problematic issues identified in their previous study. Both studies focused on the usability, acceptability, and feasibility of the Attention Training Application. The experimental design of the study in 2018 was very similar to the pilot study in 2015. Before the start of exercise, 2 preliminary sessions were established: in the first session an examiner met with a participant in order to introduce them to the device (iPad or iPad Mini). Prior to the beginning of the trial, participants could exercise using the device for a week to become familiar with its functioning. The app was then introduced in the second session. Written instruction was given to participants, and they were able to contact the experimenter to receive further information and instructions on the use of the app and device. This app delivered a program targeting attention and was based on the dual n-back training paradigm. This cognitive domain was improved by different exercises: in the first one a visual stimulus was presented, in the second one an auditory stimulus was given, and in the third step visual and auditory stimuli were presented together. The visual stimulus was a grid split in 8 parts with a square in 1 of them. The auditory stimuli were spoken letters. Given that the authors follow the n-back paradigm, the exercise requested subjects to report the correct answer of the exercise presented n trial before. At the end of each trial, the app sends a visual stimulus so the participants know immediately if the answer is the correct one. The Attention Training Application was adaptive: if participants performed well, the exercise increased in difficulty. The differences between the 2 versions of the app consisted mostly in modified elements to help the user better comprehend how to use the app and correctly perform the exercise. In 2018, the authors added some preliminary training features: a first session with the experimenter that presented the iPad and a week to become familiar with the device. Moreover, feedback was modified in the second version. While the 2015 app gave negative feedback to users (a red X indicated each wrong answer), this was removed in 2018 because participants generally reported frustration and confusion with it. Finally, the response time changed from 3 seconds to 5 seconds. The paradigm followed, and the exercise and adaptive style of exercise presentation remained the same.

Lu and colleagues [62] developed a prototype of an app called Brain Win. The app was directed to older people and evolved through 2 cycles of design and evaluation. The app involved 4 tasks and 6 games to train 5 cognitive domains: attention, executive function, memory, language, and visuospatial function. The 6 implemented games directly relate to real-life experience and daily activities of older adults:

1. My Calendar: discrimination task to train attention, executive function, and memory. Participants identify the correct date and time.
2. Go to Market: visuomotor task to train attention, executive function, memory, and visuospatial function. Participants draw the route to the market place.
3. Shopping in the Market: calculation task to train attention, executive function, and memory. Participants buy items with a limited budget and make calculations using the item prices.
4. Finding Objects During a Phone Call: discrimination task to train attention, memory, language, and executive function. Participants listen and find correct items on the screen.
5. Super Singer: respelling task to train attention, memory, language, visuospatial function, and executive function. Participants reorganize character cards with song lyrics, listening to or reading them.
6. Go to the Zoo: discrimination task to train attention, memory, and executive function. Participants recognize and remember the noise made by the zoo animals and identify them.

Each game was supported by visual and auditory instructions. Buttons and icons had a realistic appearance and participants were asked to test the app in their home. Feedback sounds were presented at the end of each task. There was also the possibility to check the game scores or participant’s position in a ranking table.

The Arean and colleagues [64] study aimed at testing the efficacy of 3 apps in relieving depressive symptomatology. These 3 apps were called Project: EVO, iPST, and Health Tips. In our review, we are mainly interested in Project: EVO, which trained cognitive control in order to improve cognitive symptoms. The other 2 apps were used to control treatment (Health Tips) and conduct psychotherapy intervention to improve depression (iPST). The participant had to use just 1 of the 3 apps: if they owned a mobile phone but not an iPad, the experimenter gave them one to use. Project: EVO is an exercise to train cognitive control and is designed as a video game. As the participants improved their proficiency with the game over time, the app increased the difficulty of the exercises. The app had an internal system to remind individuals to complete the exercise.

Blackburne and colleagues [69] tested the efficacy of the NoGo app that considered cognitive restraint—and specifically inhibitory control—to be used for weight control in obese adults. In particular, the app trained 3 domains: unhealthy eating, smoking, and alcohol consumption. Each game had 2 distinct tasks: Go and NoGo trials, in which a timer appeared next to an image that remained the same and started to countdown, and the Stop trial, in which participants had to choose the healthy food image as fast as possible. In this exercise, the image changed from healthy to unhealthy after the countdown began. Participants had to tag the correct answer if the Go tone was produced; if the NoGo tone was reproduced instead, participants had to hold back from answering. The stimuli order changed in each game so that the individuals could not anticipate and predict the next image and could not respond based on their previous experience with the game. The difficulty level was modulated by the timer and by the number of images presented (with a maximum of 12 images). The app collected data such as reaction time, game level, correct responses, and errors. The researcher could gain access to the data at the end of the training session.

Powell and colleagues [65] aimed at developing and testing an app called ProSolv, a problem-solving app developed by the authors after focus groups and interviews. In this study, the device was not provided; an inclusion criterion was that each participant must own a mobile phone and have access to the internet. The ProSolv program included 3 steps plus FAQ and help pages. The first was a face-to-face meeting with a coach; during this session, each participant could learn and test the app with the help of the expert and the program manual. In the second, a Web-based tutorial introduced the conceptual model of problem solving and its usefulness in everyday activities. Next, use of the app was explained in a video. The third step consisted of participants using the app to create a problem-solution list and remember each step to solve problems in a more effective way. The app comprised 4 pages: Welcome to the ProSolv app, My problem, My solution, and My contact. With the app, individuals could evaluate each problem-solution on the list by rating it with 1 to 5 stars.

Tacchino and colleagues [66] described the Cognitive Training Kit (COGNI-TRAcK). In their study, the app was used as a cognitive rehabilitation intervention based on working memory exercises in a sample of patients with multiple sclerosis. The authors stated that COGNI-TRAcK could be used to implement 3 types of working memory. The first task targeted visuospatial working memory and presented a sequence of visual stimuli, with participants asked to touch the corresponding location where the stimuli had appeared on the screen. The second task was an operation n-back exercise in which participants were presented with 2 numbers on the screen. If the instruction on the screen said N=0, participants had to touch the sum of the 2 numbers as the right answer. When the instruction said, for example, N=1, they had to touch on the screen the sum of the numbers previously presented. The third was a dual n-back exercise in which each patient was presented with a single number on the screen. Similar to the first n-back task, if the instruction said, for example, N=1, participants had to remember and recall the number presented in the previous exercise by touching it on the screen. All the information collected by the app was directly stored in a database with 3 sections: (1) Patient contained participant data; (2) Exercise and Treatment contained information about the assignment, workload, record of the exercise, and length of the intervention; and (3) Setting contained the characteristics of the configuration of the app. The main feature of the app was the possibility to implement the workload and regulate its intensiveness.

The study by Zmily and colleagues [68] focused on the usability of a subtask of an integrated app named ADcope. The app was developed for mobile devices and aimed to support Alzheimer disease patients in their daily routines. During the study, patients were given a tablet with the app along with clear instructions on how to use the device and app. Patients were asked to perform exercises while sitting together in a room. The app had sections for improving user quality of life and a support module, but for our review, we focused on the third section, which aimed to exercise patient memory in 2 ways. Audio-Assisted Memory Training played audio files of patient biographical information and then quizzed them about the information. The Spaced Retrieval exercise, whose usability was the main focus of the study, comprised 2 phases. The first assessed users’ current memory recall ability by presenting them with information followed, after some delay, by a question with 4 multiple choice answers. This exercise could use text information or a simple figure. The difficulty of the exercise could be operationalized by measuring the length of the delay between the information and the quiz. If the individual gave the right answer after a certain period of time, the app would increase the difficulty by increasing the time delay. The training phase (the same as the assessment) comprised 10 questions with a delay length based on the assessment results. After the participant completed the trials, written feedback appeared on the display. The app also has text-to-speech tools enabling patients to have the information read aloud.

### Cognitive Domains

#### Overview

As shown in Table 1, the cognitive domains trained by the identified apps are attention, problem solving, memory, cognitive control, executive function, visuospatial function, and language. Nine out of 11 studies considered the usefulness of the app for the training of just 1 cognitive domain at a time (cognitive control [64,69], attention [60,61,67], language [59], problem solving [65], and memory [66,68]). The remaining 2 studies considered more than 1 cognitive function: Lu and colleagues [62] investigated attention, memory, executive function, visuospatial function, and language; Shellington and colleagues [63] investigated memory, executive function, and visuospatial function.

#### Cognitive Control

Cognitive control was trained in studies by Arean and colleagues [64] and Blackburne and colleagues [69]. Both of these authors stated that app usage could improve cognitive control as a mean to enhance other conditions: depression for Arean and colleagues [64] and obesity for Blackburne and colleagues [69]. Cognitive control in the paper by Arean and colleagues [64] was trained with an app that is designed as a video game that modulates cognitive control abilities. Blackburne and colleagues [69] instead reported the efficacy of cognitive control training in food consumption in an obese population.

#### Problem Solving

People with cognitive impairment following brain injury often lack problem-solving skills, and a Web-based approach could be useful in rehabilitation [65]. The ProSolv app from Powell and colleagues [65] could be a useful tool to help solve this issue. The deficit in problem-solving was trained through the creation of a personalized problem-solution list and with the possibility to use the app as a resource for remembering the steps to effective problem solving.

#### Memory

This cognitive dimension was analyzed in different samples and with different meanings by the authors. Tacchino and colleagues [66] and Zmily and colleagues [68] explored how to train memory in patients with diseases that cause cognitive impairments: multiple sclerosis and Alzheimer disease, respectively. As they referred to different kinds of populations, the authors explored different kinds of memory. Tacchino and colleagues [66] focused on working memory training, which can lead to changes in a healthy individual’s brain structures that can improve cognitive function, useful in the daily life. The improvement is demonstrated in cognitively impaired patients as well. Their study focused on multiple sclerosis, as it is a cognitively disabling condition and affects all age groups. Another disease that has serious effects on cognitive impairment is Alzheimer disease. Memory impairment in Alzheimer disease patients was the focus of the work of Zmily and colleagues [68], which focused on recall ability training to help individuals retain critical information longer and consequentially improve their quality of life.

The research of Lu and colleagues [62] and Shellington and colleagues [63] investigated memory impairment in older populations. Shellington and colleagues [63] considered older adults with and without subjective cognitive complaints and mild cognitive impairment. In the study, the authors tried to train memory through physical activities. They stated that physical exercise was associated with higher cognitive function and used a square step exercise that was proven to train memory skills. Lu and colleagues [62] studied age-related memory decline and its effect on recollection ability during information finding and retrieval in healthy older adults. Memory was trained with 4 tasks: discrimination, visuomotor, respelling, and calculation.

#### Executive Function

Lu and colleagues [62] reported some evidence on age-related decline in executive function and performance; these abilities are generally affected by decreases in working memory functioning and by the perception of time. Deficiency in this domain is related to future functional impairment. Neurochemical, localized, and process aging theories indicate that age-related cognitive changes also affect executive functioning. Brain Win, the app developed by authors, stimulated executive function in all game contexts with 4 types of tasks (discrimination, visuomotor, respelling, and calculation). Shellington and colleagues [63] studied executive function related to physical exercise. They considered physical activities as a means to train executive function. Their app, Health*e* Brain, suggested a series of exercises, called square step exercises, to implement the cognitive domain.

#### Visuospatial Function

Brain Win, the app developed by Lu and colleagues [62], stimulated the visuospatial function with 2 tasks: Go to the Market and Super singer. Brain Win was specifically developed to improve visuomotor ability in older adults; visuospatial function is affected by age-related cognitive changes including visuospatial attention, memory, and orientation decline. Visuospatial function is the focus of the work by Shellington and colleagues [63] as well. Their study combined physical activities with cognitive training. In particular, they focused on a series of activities called square step exercises. These exercises could be described as a visuospatial working memory with a cued stepping response also known as mind-motor exercise.

#### Attention

Four studies considered the cognitive domain of attention. In particular, Bless and colleagues [67] focused on an app to train auditory attention, based on the forced-attention conditions of the consonant-vowel dichotic listening paradigm. Individuals have to listen to different auditory stimuli simultaneously in both ears while paying attention to only 1 of the sounds. According to the authors, this paradigm could be considered as an analog task in some everyday life situations in which people are asked to effectively master different and confounding auditory events. The authors reported that some deficits in auditory attention could also be found in clinical conditions like schizophrenia, preterm–born adolescents, dyslexia, and aging. In their study, the authors tried also to see if the trained task has transfer effects on cognitive interference and attentional task in daily visual and auditory domain activities.

The Attention Training Application by Hill and colleagues [60,61] is based on the dual n-back training paradigm and targets attention in elderly people. The authors reported that through aging, some aspects of attention could tend to decline—for example, with increasing age, the ability to divide or switch attention could decline. Attention is a cognitive function that has a pervasive influence on several daily activities. Thus, its training may also lead to improvements in other cognitive performance aspects and enhance the appropriateness of several daily life activities. Specifically, the authors highlighted that the effect of the dual n-back task could be transferred to other cognitive abilities.

Also, the Brain Win app, developed in a working prototype for older adults by Lu and colleagues [62], targets the cognitive domain of attention. This app directly stimulates attention with 4 types of games addressing discrimination, visuomotor, respelling, and calculation tasks.

#### Language

Two studies directly focused on training the cognitive domain of language. Lu and colleagues [62] started by reporting evidence of a decline of language functioning in elderly people. Specifically, neurochemical, localized, and process aging theories demonstrate age-related cognitive changes affecting language. Language is connected with various cognitive aspects; a decline in this domain could also affect sentence understanding and text recalling. In Brain Win, the app developed by the authors, language is stimulated through 2 context games (Finding Objects During a Phone Call and Super singer) that were connected to 2 types of abilities, discrimination and respelling, respectively. Lorusso and colleagues [59] focused on improving language ability in children with language impairment and typically developing children. Their system aimed to improve semantic competence and structural knowledge with various activities. The system is made up as an integrated combination of a tablet, a group of plastic toys, the near field communication technology, and a custom app that allows children to play with various activities to train specific cognitive processes and abilities such as mental representation, conceptual networks, and semantic versus structural world knowledge. These aspects are especially relevant in speech and language development and functioning.

### Efficacy

Only 3 out of 10 studies directly evaluate the apps in improving cognitive abilities. Blackburne and colleagues [69] evaluated the efficacy of the NoGo app in having an impact on cognitive control and in particular on inhibitory control. Compared to a control group, the authors found in people using the app an increase of cognitive restraint evaluated with a self-report questionnaire. Regarding the exercise conveyed by the app, authors showed a significant effect in improving inhibitory control in the training group. Other measured outcomes were self-reported food consumption and attitudes toward food and diet. Results demonstrated the effectiveness of the app in improving even these aspects.

Bless and colleagues [67] concluded that the training of auditory attention through an app is feasible and successful. After 21 days of practicing, participants were found to have an increase in auditory attention. This is supported by the fact that the training group showed an increase in the performance of the exercise itself and in brain activation measured with functional magnetic resonance imaging.

Finally, Powell and colleagues [65] focused on the efficacy of their app and evaluated its effectiveness in improving problem-solving ability, but no significant effects of the cognitive training were found in comparing an intervention group using ProSolv and a control group performing traditional cognitive training. Other authors focused attention on subjective evaluations of their apps [59,61-63,66,68], but these studies lack of data regarding the effects produced by the apps on cognitive abilities.

## Discussion

### General Considerations

This systematic review provides a useful, clear, and comprehensive overview of the current state of the art in available apps for cognitive training. However, this work also raises some critical issues regarding the application of this kind of training in clinical and research practice for breast cancer patients. While some issues are broader and relevant to the field of cognitive training apps, others are more specific and directly related to their application in a cancer patient population.

### General Issues in Cognitive Training Apps

The majority of the articles included in this review have the primary aim of evaluating app usability, acceptability, feasibility, and user satisfaction; only a few of them directly focus on demonstrating efficacy in improving cognitive functioning. Only 2 [67,69] out of 11 studies report significant and quantitative amelioration in cognitive performance. Even if feasibility is important, it should be noted that the objective efficacy of the apps has not been measured in the majority of the relevant articles. For several apps, we could not establish whether the training effectively improved the targeted cognitive domains.

In addition, some definitional and conceptual issues about cognitive functions also emerge. Specifically, there is a vast heterogeneity in the definition and conceptualization of cognitive function across studies, with similar cognitive domains being labeled in different ways (eg, executive functions may include visuospatial attention, attention, and so on). Moreover, targeted cognitive functions are not unique or consistent across studies, with only marginal overlap between studies. Some cognitive functions (eg, memory) cover heterogeneous abilities (eg, working memory, short-term memory, long-term memory) encompassing specific functions that can be selectively impaired in some diseases and not in others. Thus, it is difficult to draw conclusions on which cognitive domain could benefit from app training. Given the aforementioned issues, it is critical to properly evaluate the clinical efficacy and effectiveness of these apps.

### Specific Issues in Cognitive Training Apps Applied to Cancer Patients

Taken together, these results show that there are apps that are best candidates to target cognitive functions generally impaired in women with breast cancer. However, the feasibility, usefulness, and efficiency of cognitive training in this particular population should not be automatically taken for granted.

Relevant articles included in our review employed highly heterogeneous samples of subjects: only a few apps have been tested on patients, such as people with Alzheimer disease [68] or multiple sclerosis [66]; most have been evaluated on a healthy general population sample of adults or children. This heterogeneity is also indicative of the different needs of these diverse populations.

People with breast cancer may benefit from using available interventions or to-be-developed apps for this specific category of patients. In fact, breast cancer patients somehow reside in a class between mildly or severely cognitively impaired patients and healthy general population subjects because they display objective cognitive impairments or report cognitive problems [9] but they still can properly work and have an active and preserved social life. On one hand, they do not display a degenerative disease with a progressive worsening of global or selective cognitive functioning. On the other hand, they are aware of and worried that their cognitive worsening could interfere with daily routines and everyday life. Thus, they are strongly motivated to be involved in a cognitive training program. Worthy of note, breast cancer patients usually display a high level of distress symptoms and serious psychological side effects [5,9] that can influence subjective perception of cognitive functioning and could potentially interfere with training activities.

Finally, the summarized papers, apart from Lu and colleagues [62] and Shellington and colleagues [63], reported training interventions on a single cognitive domain. However, women with breast cancer often display difficulties and deficits in several cognitive domains. Thus, it could be relevant to design, develop, and implement an ad hoc app targeting the various cognitive function domains for breast cancer patients.

### Conclusion

Our study highlights the fact that cognitive training apps are becoming more present in rehabilitation of different diseases. It is noteworthy that none of them has been developed to counteract cognitive impairment in breast cancer patients, a specific population in which short- and long-term cognitive difficulties have been underlined. Thus, currently there are no available cognitive training apps that meet the needs of breast cancer survivor women. Available apps lack strong specificity for oncological breast patients both from the point of view of the cognitive functions that should be addressed and for the psychological complexity that these patients display. In fact, the psychological and physical impact of breast cancer on cognitive impairment should be taken into account as well. As a specific population with specific needs, it is necessary to create an app that considers their deficit as different from the deficit that another population of patients could have (eg, neurodegenerative conditions). Medicine is evolving to consider not only the patient’s physical safety but also a personalized approach to disease [70,71]. From the patient empowerment perspective, it is very important to give breast cancer survivors reliable means to improve and train their cognitive functioning because of the huge impact of cognitive complaints on the quality of life and patient empowerment [72]. Moreover, women with breast cancer may benefit from using a mobile or Web-based tool to improve cognitive functioning, effectively manage their daily activity, and properly cope with everyday difficulties. This would be especially helpful to foster breast cancer patient perceived self-efficacy and manage their anxious and depressive symptomatology. We conclude that further studies should test the feasibility, usability, and effectiveness of available cognitive training apps in women with breast cancer. Because of the complexity and multidimensionality of the cognitive difficulties affecting this cancer population, it may be useful to design, develop, test, and implement an app with the specific aim to train cognitive impairment in breast cancer patients.

## Appendix

Multimedia Appendix 1 Summary of study characteristics in terms of study type, aim, and sample.

Multimedia Appendix 2 Summary of main aspects of apps and their evaluation.

## Abbreviations

COGNI-TRAcK: Cognitive Training Kit
