# Supplementary material for: Training Cognitive Functions Using Mobile Apps in Breast Cancer Patients: Systematic Review
Source: JMIR Mhealth Uhealth. 2019 Mar 19;7(3):e10855. doi: 10.2196/10855 (PMC6444278; doi:10.2196/10855)
Supplement: Multimedia Appendix 1 [file mhealth_v7i3e10855_app1.pdf]

Multimedia Appendix 1. Summary of study characteristics in terms of study type, aim and sample.

Authors	Aim	Sample			
		N	Age	Country	Population Type
Arean and colleagues, 2016 [24]	To test outcome and clinical patterns of three different mobile apps for depression	626	Mean: 33.9<br>SD:11.8<br>Range: -	USA	Adults with mild to moderate depression
Blackburne and colleagues, 2016 [25]	To examine the efficacy of a training programme for inhibitory control	52	Mean: 35.2<br>SD: 14.1<br>Range: 19-61	Australia	overweight or obese adult.<br>- training participant,<br>-waitlist/ control
Bless and colleagues, 2014 [26]	To test the feasibility of an app for training attention	28	Mean: 23.3 e 23.9<br>Range: -	Norway	Healthy individuals<br>-non-training control group<br>-training control group
Hill and colleagues, 2015 [27]	To test the app feasibility	9	Mean: 76,1<br>SD: 5.5<br>Range: 64-96	USA	Old Adults
Hill and colleagues, 2017 [28]	to evaluate the usability and acceptability of an app to train attention	12	Mean: 79<br>SD: 4.2<br>Range: -	USA	Old adults
Lorusso and colleagues, 2017 [29]	to evaluate learnability, usability, user satisfaction and quality of the interaction between an app to train language and children	14	Mean: 60.1 Month<br>SD: 6.2<br>Range: 50-68 month	Italy	Children with mild to severe language impairments or delays
Lu and colleagues, 2017 [30]	to develop an improved design for game-based cognitive training for seniors using mobile devices.	9	1Mean = 82.7<br>2Mean = 69.6<br>SD1 = 7.2<br>SD2 = 9.4 1= 4 e 2= 5<br>Range1: 73-90<br>Range2:61-84	Taiwan	Old Adults
Powell and colleagues, 2017 [31]	To develop and test an app to train problem-solving in everyday life for patients following brain injury.	23	Mean: 44<br>SD:15<br>Range: 20-75	USA	Brain injuries in adults.<br>-Control group,<br>-Intervention
Shellington and colleagues, 2017 [32]	to explore the feasibility and utility of an app to deliver physical exercise outside the laboratory	20	Mean: 68<br>SD: 5.4<br>Range: 59-76	Canada	Older adults with and without Subjective Cognitive Complaints (SCC) and Mild Cognitive [illegible]
