# Supplementary material for: Training Cognitive Functions Using Mobile Apps in Breast Cancer Patients: Systematic Review
Source: JMIR Mhealth Uhealth. 2019 Mar 19;7(3):e10855. doi: 10.2196/10855 (PMC6444278; doi:10.2196/10855)
Supplement: Multimedia Appendix 2 [file mhealth_v7i3e10855_app2.pdf]

Multimedia Appendix 2. Summary of main aspects of apps and their evaluation.

Authors	Domain Trained	Exercise Description	Assessment And Feedback	Evaluation		Training With Experimenter	Device Given
				Subjective Evaluation	Efficacy		
Arean and colleagues, 2016 [24]	Cognitive Control	Created as a video game	-	Half individuals downloaded the app. App used 10.78 times	Not evaluated	-	-
Blackburne and colleagues, 2016 [25]	Inhibitory Control (Cognitive Control)	3 components of inhibitory control: -unhealthy eating, -smoking, -alcohol consumption. Training task is based on "Go/NoGo and stop signal task".	X	Not evaluated	Improved inhibitory control in training group	X	-
Bless and colleagues, 2014 [26]	Auditory Attention	Training task is based on the "forced-attention conditions of the consonant-vowel dichotic listening paradigm"	-	Not evaluated	Better performance and lower activation in brain in the training group	-	X
Hill and colleagues, 2015 [27]	Attention	The training task is based on three parts: presentation of visual stimuli alone, presentation of auditory stimuli alone, a combination of visual and auditory stimuli	-	Low feasibility	-	X	X
Hill and colleagues, 2017 [28]	Attention	The training task is based on three parts: presentation of visual stimuli alone, presentation of auditory stimuli alone, a combination of visual and auditory stimuli	-	Positive usability, interest, enjoyment, and satisfaction. Difficult to use the app alone	Not evaluated	X	X
Lorusso and colleagues, 2017 [29]	Language	Training task is based on activities to improve [illegible]	-	Easy to understand and learn, engaging and [illegible]	Not evaluated	X	X
